# Anthropometric Measurements in Newborns: A Comparative Study of Infants Born to Mothers With and Without Polycystic Ovary Syndrome

**DOI:** 10.7759/cureus.48012

**Published:** 2023-10-30

**Authors:** Jayakumari S, Nirupa S, Shivaranjani K S, Geetha Haripriya, Dhastagir Sheriff, Janaki C S, Hassan Mohammad, Prabhu K

**Affiliations:** 1 Anatomy, Sree Balaji Medical College and Hospital, Chennai, IND; 2 Obstetrics and Gynaecology, Sree Balaji Medical College and Hospital, Chennai, IND; 3 Obstetrics and Gynecology, Sri Lalithambigai Medical College & Hospital, Chennai, IND; 4 Obstetrics and Gynecology, Prashanth Fertility Research Centre, Chennai, IND; 5 Biochemistry, Anna Medical College, Montagne Blanche, MUS; 6 Anatomy, Bharath Medical College and Hospital, Chennai, IND; 7 Anatomy, Northern Border University, Arar, SAU

**Keywords:** ponderal index, large for gestational age (lga), small for gestational age (sga), anthropometric measurements, polycystic ovary syndrome (pcos)

## Abstract

Objective

Fetal growth can be affected by maternal PCOS and may have an impact on offspring and childhood growth. The current findings across studies are divergent and controversial. This study aims to determine whether maternal PCOS can affect the physical measurements of newborns and to establish the differences in birth weight, length, head, and chest circumference between newborns of mothers with polycystic ovarian syndrome and those of mothers without polycystic ovarian syndrome.

Methods

In this study, we examined the gestational age, birth weight, length, head circumference, chest circumference, and ponderal index of 75 infants born to mothers with polycystic ovary syndrome (PCOS) and compared them to those of 94 infants born to mothers without PCOS.

Result

Compared with the other groups, the PCOS group does not show significant differences in anthropometric indices compared to the control group. Infants born to normal and PCOS mothers birth weight were categorized as SGA (small for gestational age) if birth weight was less than the 5th percentile. LGA is large for gestational age if birth weight is greater than the 90th percentile. Other appropriate for gestational age if infant birth weight is between> 5th and < 90^th^ percentile. Significant differences in anthropometric indices like birth weight, head circumference, and Ponderal index were observed in SGA and LGA newborns of normal and PCOS mothers.

Conclusion

The study findings indicate that neonates born to mothers with PCOS have higher rates of SGA and LGA newborns, and differences in anthropometric indices (birth weight, head circumference, and Ponderal index) were observed between SGA and LGA newborns of PCOS mothers.

## Introduction

Polycystic ovarian syndrome (PCOS) is an endocrine disorder affecting globally 5%-10% of women globally [1]. It is associated with irregular cycles, chronic anovulation, insulin resistance, infertility, and hyperandrogenism. Obesity is one of the most common features of PCOS; however, lean women are also affected by this disease. Several environmental and genetic factors play an important role in the development of PCOS. It is also associated with an increased risk of complications during pregnancy. At the end of the first trimester, women with PCOS may encounter pregnancy complications like gestational diabetes mellitus (GDM), pregnancy-induced hypertension (PIH), preeclampsia, preterm delivery, and the birth of small for gestational age and large for gestational age infants [2-4]. A risk factor for developing obesity, PCOS, and being overweight later in life is either having a small for gestational age (SGA) or large for gestational age (LGA) birth [5,6]. With certain studies finding a higher risk of both SGA and LGA and others reporting no effects on birth weight when corrected for gestational age, the effect of PCOS on fetal growth is rather ambiguous [7].

Through this study, an effort has been made to shed more light on the typical maternal diseases that influence alterations in different anthropometric measurements of newborns and their clinical correlates. In our study, PCOS was taken into consideration. PCOS is a condition that may affect neonatal health. Therefore, the current study is aimed at determining the influence of anthropometric parameters in newborns with PCOS and normal pregnant mothers in Indian subcontinent regions.

## Materials and methods

A study was carried out on 169 mothers and their live newborn babies between 2018 and 2020 at hospitals located in and around Chennai in the Department of Obstetrics and Gynecology. The study protocol was approved by the institutional ethical board, and written informed consent was obtained from all participants before evaluation.

The study participants were divided into two groups. Group I: consists of normal women and their newborn children. Group II: PCOS women and their newborn babies. 

Group I: Ninety-four healthy women and their newborns were chosen for the study. The women were selected based on their regular menstrual cycles and the absence of clinical signs of hyperandrogenism. Subjects with congenital anomalies, preeclampsia, and hypertension were excluded. 

Group II: In this study, we focused on a group of 75 women with polycystic ovary syndrome (PCOS) and their newborn babies. The Rotterdam criteria [8] were utilized to identify these subjects, which included women with fewer than eight menstrual cycles per year, a free androgen index (FAI) greater than five, and clinical indications of hyperandrogenism such as hirsutism and acne. We excluded mothers who had hypertension, preeclampsia, or a smoking habit. Likewise, we excluded newborns who had significant congenital anomalies and those who experienced intrauterine growth retardation (IUGR).

In this study, pregnant women were followed up in the same prenatal unit, and data on the duration of gestation, initial body mass index (BMI), BMI in the third trimester, and weight gain during pregnancy were recorded. Following delivery, a pediatrician conducted a physical examination of the newborn, using standard anthropometric methods to measure length, weight, head circumference, and chest circumference. 

The subjects were considered for the study, irrespective of gestational age.

Rohrer’s Ponderal Index is a measure of body size that is calculated by dividing the weight in kilograms of an individual by the cube of their height in meters.

Instruments used

The newborn's birth weight was measured within 12 hours of birth using an electronic balance that had a margin of error of ±10 grams. To measure the crown-heel length, the baby was positioned in a supine position with knees fully extended and soles of feet held against the footboard, while an infantometer was used to record the length to the nearest 0.1 cm. The head circumference was measured by placing a non-elastic tape over the occipital protuberance on the back and supraorbital ridges in front. The chest circumference was measured at the nipple level using the same non-elastic tape. The Ponderal index was calculated.

Statistical analysis

The data analysis was performed using SAS (Statistical Analysis Software), and statistical significance was determined by a p-value less than 0.05. The mean and standard deviation were used to express normally distributed data, while the median and interquartile ranges were used for non-normally distributed data. The normality and student's T-test were used to compare data between normal mothers with polycystic ovary syndrome (PCOS) mothers and their newborns.

The Mann-Whitney test was employed to compare differences in large for gestational age (LGA) and small for gestational age (SGA) between normal newborns and those born to mothers with PCOS. Additionally, a correlation analysis was conducted between the normal and PCOS groups for variables such as initial body mass index (BMI) and BMI in the third trimester. The weight and length of the infants were also compared between the PCOS and normal groups. Furthermore, correlations were observed between gestational age in weeks and head circumference, chest circumference, and Ponderal index for both PCOS and normal groups.

## Results

Table 1 presents the clinical features of pregnant women, which were divided into two groups: normal pregnant women and pregnant women with PCOS. Age and initial BMI were not statistically different, and significant weight gain was observed between the two groups (P< 0.01).

**Table 1:** Clinical characteristics of normal pregnant women and pregnant women with polycystic ovarian syndrome (PCOS). Values are means ± SD. *P< 0.01 between normal pregnant and polycystic ovarian syndrome (PCOS) pregnant women.

Parameter	Normal (n꞊94)	PCOS (n꞊75)
Age (years)	27.78 ± 4.42	27.34 ± 3.94
Height (cm)	154.76 ± 9.5	152.18 ± 8.7
Initial weight (kg)	59.74 ± 8.49	60.22 ± 10.43
Initial BMI	25.25 ± 5.1	26.55 ± 4.8
Third trimester weight (kg)	73.69 ± 10.13	76.12 ± 11.87*
Third trimester BMI	31.21 ± 6.4	35.21 ± 5.8*
Weight gain during pregnancy (kg)	13.29 ± 4.4	16.02 ± 4.9*

Table 2 illustrates the clinical data of newborn babies from controls and PCOS mothers. No differences were observed in birth weight, birth length, head circumference, chest circumference, or Ponderal index between the two groups, and the proportion of SGA newborns in PCOS mothers was higher than the observed newborns in control mothers.

**Table 2:** Clinical characteristics of newborns of normal mothers and polycystic ovarian syndrome (PCOS) mothers. Values are means ± SD.

Parameter	Normal (n=94)	PCOS (n= 75)
Gestational age (weeks)	38.32 ± 1.41	38.44 ±1.31
Birth weight (kg)	2.84 ± .5	3.14 ± .44
Length (cm)	51.80 ± 4.5	49.36 ± 3.5
Head circumference (cm)	33.64 ± 2.3	34.34 ± 2.8
Chest circumference (cm)	31.14 ± 2.2	31.39 ± 4.2
Ponderal index	21.4 ± 6.9	22.6 ± 5.5

According to the data presented in Table 3, increased weight and BMI were observed in the third trimester of PCOS mothers, which is statistically significant. 

**Table 3:** Small for gestational age (SGA) infant mothers' data compared normal mothers and PCOS mothers. Values are means ± SD. *P< 0.01 between normal pregnant and polycystic ovarian syndrome (PCOS) pregnant women.

Parameter	Normal (n=10)	PCOS (n=6)
Height (cm)	151.60 ± 8.50	154.83 ± 12.25
Initial weight (Kg)	61.10 ± 7.07	59.08 ± 6.65
Initial BMI	27.64 ± 5.52	26.73 ± 3.76
Third trimester weight (Kg)	75.00 ± 8.32	79.75 ± 10.69*
Third Trimester BMI	32.77 ± 4.09	35.92 ± 8.15*
Weight gain during pregnancy (Kg)	13.90 ± 3.6	16.66 ± 6.74*

Table 4 displays that birth weight is lower and head circumference and Ponderal index are higher in the PCOS group, which is statistically significant.

**Table 4:** Clinical characteristics of small for gestational age (SGA) newborns’ data compared with normal mothers and PCOS mothers. Values are means ± SD. *P< 0.05 between normal pregnant and polycystic ovarian syndrome (PCOS) pregnant women.

Parameter	Normal (n=10)	PCOS (n= 6)
Gestational age (weeks)	35.50 (33 to 36)	33.17 (35 to 36)
Birth weight (kg)	2.60 (2.1 to 3.5)	2.23 (2.1 to 3.5)*
Birth length (cm)	53.40 (48 to 63)	47.75 (43 to 50)
Head circumferences(cm)	32.32 (30.1 to 38.1)	33.81 (26.5 to 37.6)*
Chest circumference(cm)	31.58 (28.1 to 36)	31.36 (24.2 to 35)
Ponderal index	17.60 (10.79 to 23.51)	24.11 (23.8 to 28)*

Table 5 presents comparable numbers of mothers delivering large-for-gestational-age (LGA) babies in both study groups. PCOS mothers demonstrated higher values for initial BMI, third-trimester weight, and BMI in the third trimester compared to normal mothers, all of whom delivered LGA babies. These findings have important implications for clinicians managing pregnancies complicated by PCOS, as they highlight the potential role of maternal weight and BMI in predicting fetal overgrowth.

**Table 5:** Large for gestational age (LGA) infant mothers' data compared with without PCOS mothers (normal) and PCOS mothers. Values are means ± SD. *P< 0.01 between normal pregnant and polycystic ovarian syndrome (PCOS) pregnant women.

Parameter	Normal (n꞊20)	PCOS (n꞊20)
Height (cm)	154.65	153.70
Initial weight (kg)	61.30	64.05
Initial BMI	26.04	27.17
Third trimester weight (kg)	76.65	79.75*
Third Trimester BMI	32.67	34.39*
Weight gain during pregnancy (kg)	13.85	15.15

Table 6 presents the findings that increased birth weight was observed in the case of LGA (large-for-gestational-age) newborns born to mothers with PCOS (polycystic ovary syndrome) as compared to those born to normal mothers. However, the measurements for head and chest circumference were found to be higher between the two groups. Moreover, a significant difference was observed in the Ponderal index of LGA babies between PCOS and normal mothers, with values of 26.01 and 21.35, respectively.

**Table 6:** Clinical characteristics of large for gestational age (LGA) newborn data compared with normal mothers and PCOS mothers. Values are means ± SD. *P< 0.05 between normal pregnant and PCOS pregnant women.

Parameter	Normal (n=20)	PCOS (n=20)
Gestational age (weeks)	40 (40 to 40)	39.97 (40 to 40)
Birth weight (gm)	2.79 (1.9 to 3.6)	3.27 (2.6 to 4)*
Length (cm)	51.85 (43 to 61)	49.80 (43 to 60)
Head circumference (cm)	34.23 (31.2 to 37.1)	35.12 (28.5 to 31.1)*
Chest circumference (cm)	31.82 (26.3 to 36.2)	31.48 (26.3 to 35.6)
Ponderal Index	21.35 (10.22 to 44.02)	26.01 (16.20 to 50.31)*

## Discussion

In this study, the incidence of both SGA and LGA newborns was higher in PCOS mothers. Statistically significant higher birth weight, head circumference, and Ponderal index were observed in SGA and LGA newborns of PCOS mothers when compared to newborns of control mothers. The weight and weight gain in the third trimester were statistically significantly higher between PCOS and control women.

In a study by Melo [7], he observed a higher incidence of SGA newborns in PCOS mothers and observed more visceral fat than those born to normal mothers. A separate study in Australia also found a higher incidence of small-for-gestational-age offspring born to mothers with PCOS [9].

According to another study conducted in Sweden [10], mothers with polycystic ovary syndrome (PCOS) give birth to more babies who are larger for their gestational age compared to mothers without PCOS. Another two studies with matched maternal pregestational BMI showed more SGA-decreased birth weight in PCOS, although another study reported a similar report regarding birth weight [11,12]. In another meta-analysis study, Qin et al. found an increased risk of lower birth weight in PCOS offspring.

In contrast, recent data illustrates that no association exists between small for gestational age (SGA) or large for gestational age (LGA) newborns and PCOS [13]. However, a retrospective study in Austria by Kollmann [14] suggests PCOS was not associated with an increased risk of growth deviation or weight gain during pregnancy in PCOS mothers.

However, the Danish cohort study found no difference in birth weight, Ponderal index, or abdominal circumference between control offspring and PCOS-offspring adjusted for maternal age, BMI, gestational age, and sex, which is also consistent with our study [15]. The offspring birth weight increases with increasing maternal pre-pregnancy BMI. An increased number of LGA babies and a higher birth weight would be expected from PCOS mothers because they are overweight, weight gain increases in pregnancy, hidden growth restriction, and altered body composition may be present in the newborns of some PCOS women despite seemingly normal birth weight. However, increased birth weight is a poor surrogate marker for a suboptimal intrauterine environment or fetal growth restriction [16].

The increased head circumference and weight of the infant at birth were observed and compared with controls in a porcine model of intrauterine growth restriction [17,18].

Head circumference at a younger age is strongly correlated with brain volume, and at a population level, a larger brain is associated both with autism spectrum disorder and better cognitive function [19,20]. Head circumference at birth is also a reliable measure of brain weight and fetal brain growth. A Danish study showed that a large head circumference at birth was associated with a decreased risk of intellectual disability [21,22]. Even so, it has also been shown that small head differences may not be correlated with differences in IQ [23].

Therefore, it may be beneficial for these individuals to receive timely interventions, such as dietary and exercise-based therapies, as an adjunct to their treatment [16].

Fetal development is a critical period of physical development that begins at conception and ends at birth [17]. During this period, the developing fetus undergoes a rapid process of growth and differentiation, during which various systems and organs are formed and functionalized. The intrauterine environment, including factors such as maternal nutrition, stress, infections, exposure to toxins, and other environmental factors, can influence fetal development and have lasting effects on the growth and development of offspring. For example, inadequate maternal nutrition during pregnancy can lead to low birth weight and increase the risk of developmental problems in SGA newborns and chronic diseases later in life. Exposure to toxins such as alcohol, tobacco, and certain drugs can also cause long-term effects on fetal development, including birth defects, cognitive impairment, and behavioral disorders. 

The primary constraints of the research revolve around the relatively small participant pool and the suboptimal response rate among those who were invited to participate. Despite the fact that the current sample size yielded statistically significant findings in the parameters under consideration, a larger number of participants would have provided a more definitive and precise depiction of the results. Another limitation encountered in this study was the inability to conduct a comparative analysis of cognitive assessments between children born to mothers with normal conditions and those with polycystic ovary syndrome (PCOS). Notably, there exists the possibility that certain children with low-to-borderline intellectual capabilities were not accounted for in the follow-up due to the overwhelming challenges experienced by both these children and their families.

Therefore, it is important for expectant mothers to take care of their health and well-being during pregnancy to ensure the best possible outcomes for their offspring. This can include maintaining a healthy diet, avoiding exposure to harmful substances, and seeking medical care when needed.

## Conclusions

The study findings indicate that neonates born to mothers with PCOS have a higher combined incidence of both SGA and LGA newborns. Moreover, a notable statistically significant higher value was observed in head circumference and Ponderal index among babies born to SGA and LGA newborns of PCOS mothers when compared to those born to normal mothers. These observations suggest that the PCOS phenotype is expressed during the neonatal period, and the impact of PCOS is unable to be evident at the clinical level in the early stages of life.
